# A New Test for Typhoid Fever

**Published:** 1897-12

**Authors:** 


### A NEW TEST FOR TYPHOID FEVER.

The tests for typhoid fever are not always reliable, and many a disease gets the name of typhoid when it is in reality something else, and of some other fever when it is really typhoid. The "rose spots" to which the books point as indications of typhoid are a good test when they exist, but they are absent in at least thirty per cent. of typhoid fever. Other "characteristic" symptoms are not always present, and when they are present they may mean malaria or something not typhoid, so that often the physician can not say positively before the tenth day whether a case is really typhoid. Not long since a test, discovered by Robert Pfeiffer, Koch's assistant, was announced. It was called the Widal system, and was very far from satisfactory, leaving the matter in about as much doubt as before.

By this test a drop of blood or serum is mixed with a pure typhoid culture. When put under the microscope, if there has been typhoid in the system within the past five years the bacilli would be seen under the lens to gather in groups, but if there had been no typhoid the bacilli would move about with freedom. This, of course, was unsatisfactory, and required great delicacy of manipulation to afford much aid in diagnosis.

It is a very excellent rule in the health department that each bacteriologist shall take up under the direction of the chief of the laboratory, Dr. Biggs, each year some new subject of research. In this way long and continued observation is centered upon one line of investigation, and every year excellent results are obtained.

Last year Dr. Hiss of the Health Department, recognizing the fact that the typhoid bacillus must be located in the intestines, and recognizing also the fact that another microbe, the colon bacillus, which is perfectly harmless, is found there also, and that even with a microscope the resemblance between them is so great that it is very difficult to differentiate between them, he attempted to determine the presence of typhoid bacillus by another method. After repeated experiments and minute study, he devised a culture in which typhoid germs will multiply and the other will not, which worked so satisfactorily that the Health Board adopted it officially.

The culture is composed of gelatine, agar (which is a Japanese isinglass much in favor with bacteriologists), glucose (to make the mixture ferment), salt and Leibig's food. Typhoid germs grow in it, and the colon bacillus does not. In actual practice a minute quantity of the semi-fluid matter sent for examination is taken off on a "loop" and is placed in a little vial containing this "culture." Eighteen hours is sufficient time for the bacilli to grow, and a statement of results will reach the doctor inside of thirty-six hours after he has sent a specimen to the laboratory. It is the plan of the Board of Health to have all the doctors of New York City avail themselves of this test free of charge. At the end of eighteen hours a small portion of the culture is spread on a glass plate which has a glass cover. In a short time, to a practiced eye, the appearance of this plate will give the information sought—that is, it shows if there has been any growth. If there is growth, it is a case of typhoid; if not, probably no typhoid exists.

The very great difficulty which has heretofore existed in certain obscure cases of differentiating between typhoid and malaria, if the recent tests are correct, will be in a measure solved by the above test in typhoid and the recognition of plasmodia in malaria.—*N. Y. Medical Times.*

